# Prediction of attendance at fitness center: a comparison between the theory of planned behavior, the social cognitive theory, and the physical activity maintenance theory

**DOI:** 10.3389/fpsyg.2015.00121

**Published:** 2015-02-11

**Authors:** Darko Jekauc, Manuel Völkle, Matthias O. Wagner, Filip Mess, Miriam Reiner, Britta Renner

**Affiliations:** ^1^Department for Sport Psychology, Institute for Sport Science, Humboldt University of BerlinBerlin, Germany; ^2^Department for Sport Science, University of KonstanzKonstanz, Germany; ^3^Max Planck Institute for Human DevelopmentBerlin, Germany; ^4^Department for Health Sciences, University of Education Schwäbisch GmündSchwäbisch Gmünd, Germany; ^5^Department for Sport Science, University of StuttgartStuttgart, Germany; ^6^Department for Psychology, University of KonstanzKonstanz, Germany

**Keywords:** maintenance, exercise psychology, physical activity, exercise, prediction

## Abstract

In the processes of physical activity (PA) maintenance specific predictors are effective, which differ from other stages of PA development. Recently, Physical Activity Maintenance Theory (PAMT) was specifically developed for prediction of PA maintenance. The aim of the present study was to evaluate the predictability of the future behavior by the PAMT and compare it with the Theory of Planned Behavior (TPB) and Social Cognitive Theory (SCT). Participation rate in a fitness center was observed for 101 college students (53 female) aged between 19 and 32 years (*M* = 23.6; *SD* = 2.9) over 20 weeks using a magnetic card. In order to predict the pattern of participation TPB, SCT and PAMT were used. A latent class zero-inflated Poisson growth curve analysis identified two participation patterns: regular attenders and intermittent exercisers. SCT showed the highest predictive power followed by PAMT and TPB. Impeding aspects as life stress and barriers were the strongest predictors suggesting that overcoming barriers might be an important aspect for working out on a regular basis. Self-efficacy, perceived behavioral control, and social support could also significantly differentiate between the participation patterns.

## Introduction

Participation in physical activity (PA) is associated with a variety of health benefits and a reduction in chronic diseases (Warburton et al., 2007). However, the benefits of PA are not maintained without continuous and regular participation. Stopping or substantially reducing PA can lead to a loss of initial health improvement (Mujika and Padilla, 2000). Empirical studies have shown that a great number of the participants in exercise programs drop out during the first six months (Dishman and Buckworth, 1996). Although important advances have been achieved to understand motivation to initiate participation in PA, relatively little research has specifically examined the maintenance of PA (Mâsse et al., 2011).

According to Rothman (2000), maintenance can be “defined as a course of action sustained over a specified period of time” (p. 65). Maintenance can be seen as a continuous process which is accompanied by variation in PA behavior over time. It is supposed that individual behavioral development is dependent on specific determinants that differ in the process of initiation and maintenance (Burton et al., 1999).

Before effective interventions can be developed the determinants of PA maintenance should be known. In this regard, theory-based interventions are more effective than approaches lacking a theoretical underpinning (Michie and Abraham, 2004). Theories are useful to provide a deeper understanding of the underlying processes, such as the role of possible mediators and moderators. Two of the most commonly used theories to explain PA behavior are the Theory of Planned Behavior (TPB; Ajzen, 1991) and Social Cognitive Theory (SCT; Bandura, 2004). In essence, both theories are relatively general and were developed to explain a broad range of behaviors. In contrast, Physical Activity Maintenance Theory (PAMT; Nigg et al., 2008) was specifically developed in order to explain PA maintenance. In the present paper, we examine whether this specific theory of PA maintenance offers a more convincing explanation of PA maintenance and compare it to the more general TPB and SCT. We start by providing a short review of the three theories:

According to the TPB, behavior is a function of a person's intention to perform behavior, which reflects the level of motivation toward performing behavior and represents the most proximal predictor of behavior. Intention is supposed to be determined by attitude, subjective norm, and perceived behavioral control. There is considerable empirical evidence that supports the applicability of the TPB to explain PA intentions and behavior (Godin and Kok, 1996; Armitage and Conner, 2001; Hagger et al., 2002). However, TPB does not distinguish between decisions regarding initiation and maintenance of behavior (Sheeran et al., 2001). It is assumed that in both phases of behavior the same constellation of predictors is efficacious. Although Sniehotta et al. (2014) propose to retire the theory of planned behavior, it provided useful theoretical framework to understand the link between beliefs and behavior and stimulated research in health psychology. There are several studies testing the TPB in the process of maintenance of PA behavior in the general adult population, which provided mixed evidence for predicting PA maintenance (Courneya et al., 1997, 2001; Bryan and Rocheleau, 2002; Armitage, 2005). For example, Armitage (2005) found that only perceived behavioral control but not behavioral intention was predictive for the attendance of a fitness center, monitored weekly for 12 consecutive weeks in a sample consisting of 94 adult participants. In general, the results of empirical studies supported the role of perceived behavioral control and behavioral intentions for the prediction of PA maintenance (Amireault et al., 2013).

Likewise, widely recognized and frequently applied in the field of PA behavior, the SCT describes factors influencing behavior (Bandura, 2004). The core set of psychosocial determinants are goals, perceived self-efficacy, outcome expectancies, facilitators, and impediments. According to Bandura (2004), self-efficacy belief is the key determinant of both initiation and maintenance of behavior. Several empirical studies showed the relevance of self-efficacy belief as a direct, and indirect predictor of PA maintenance (e.g., Burton et al., 1999; McAuley et al., 2003; Plotnikoff et al., 2008; White et al., 2012). However, the reported studies used different constructs when examining the SCT. Some studies excluded important constructs of the SCT (e.g., goals or outcome expectancies) whereas other studies included constructs not proposed by the SCT (e.g., emotional stress or health status). An exception is the study by Plotnikoff et al. (2008) with 1717 adult participants with type 1 and type 2 diabetes, which provided an excellent model fit and evidence for predictive power of the SCT in the process of PA maintenance.

The PAMT can be seen as further development and a specification of the SCT in the context of the physical activity maintenance process. Considerably less attention was devoted to the PAMT which was originally developed as a theoretical framework for supporting PA maintenance interventions. Nigg et al. (2008) acknowledged that PA initiation and maintenance have different predictors and focused explicitly on PA maintenance. As key determinants and mediators of PA maintenance goal setting, self-motivation, and self-efficacy were taken into consideration. In this theory, goal-setting is task oriented and related to behavior through satisfaction, attainment, and commitment to goals. Self-motivation represents a generalized persistence tendency to implement behavioral goals independent of beliefs about reinforcement history, ability, or control. In line with Bandura (1997), self-efficacy represents the confidence in one's own personal abilities to perform target behavior whereas Nigg et al. (2008) distinguish between barrier and relapse self-efficacy. A reciprocal relationship between the three variables is supposed to exist—nonetheless each variable has a direct unique effect on PA maintenance. Furthermore, Nigg et al. (2008) suppose supportive environment to have a positive impact, and life stress to have a negative impact on goal setting, self-motivation, self-efficacy, and PA maintenance.

To our knowledge, to date there is no empirical study testing the assumptions of the PAMT. However, the single components of the PAMT have been empirically tested. The effectiveness of self-efficacy as a predictor of PA maintenance has been shown above. In several studies, goal setting was also shown to be a significant predictor of PA maintenance (e.g., Annesi, 2002a; Greaves et al., 2011; Pearson, 2012). There is ample empirical evidence for positive effects of self-motivation on PA maintenance (e.g., Annesi, 2002b; Motl et al., 2003; Levy et al., 2008; André and Dishman, 2011). Furthermore, PA environment (Duncan et al., 2005; Wendel-Vos et al., 2007; Saelens and Handy, 2008) as well as life events (Allender et al., 2008; Engberg et al., 2012) seem to be persistently associated with PA participation. Presumably, PAMT should provide best predictions as this theory was specifically developed to promote physical activity maintenance.

The literature on determinants of PA maintenance reveals several limitations. First, a great part of the reviewed studies lack theoretical underpinning and separately analyze the determinants. This shortcoming impedes an understanding of the mechanisms at work. Second, there are only a few longitudinal studies with more than two measurement occasions, which are necessary for the analysis of PA patterns over time (cf. Armitage, 2005). Therefore, there is limited evidence for the proposed theories in predicting the behavioral trajectories. Third, most studies used self-report and retrospective data of PA. Forth, there are few studies that directly compared theories of health behavior (e.g., Carmody et al., 1980; Dishman, 1982; Dzewaltowski et al., 1990; Kimiecik, 1992; Marcus et al., 2000) in order to select best strategies for increasing PA levels. Finally, to our knowledge, there is no study that compared the theories of health behavior in the maintenance process so far. Especially, the merit of new theories, like PAMT, which explicitly address the maintenance process, should be evaluated in comparison with well-known and well-established theories of health behavior like SCT and TPB.

The purposes of the present study were (i) to describe the development of PA attendance, (ii) to predict PA participation patterns by the TPB, SCT and PAMT, and (iii) to compare the predictive power across the three theories. We assume that the predictive power of the PAMT will be superior to the TPB and the SCT as this theory is specified on two levels: (a) specified for the context of physical activity and (b) specified for the maintenance process as compared to the TPB and the SCT.

## Methods

### Participants and procedure

Participants were 101 (48 males and 53 females) college students and members of a fitness center. Age ranged from 19 to 32 years with an average age of 23.6 years (SD ± 2.9). Fitness center membership and, consequently, the possession of a magnetic card for the devices in the fitness center were inclusion criteria for this study. One week in advance of the start of this observational study, all members of the fitness center were informed by email about the study. A questionnaire measuring socio-demographic and psychological variables was distributed in the first two weeks of the semester. Participants were asked to complete the paper-and-pencil-questionnaire directly at the information center which is located at the entrance of the fitness center. Fitness center attendance was registered electronically for 20 consecutive weeks. Ethical approval for the study was provided by the University and all participants signed an informed consent form at the beginning of the study.

### Measurement

#### Physical activity participation

Participation frequency was assessed electronically by a magnetic register system which was activated when the participants used the machines. Each participant had to use the magnetic card when using the fitness center. The data were summarized as the frequency of the weekly participation. The week was defined from Monday to Sunday. The maximal score for 1 week could be 7 (when a participant visited the fitness center every single day of the week) and the minimal score 0 (when a participant did not even visit the fitness center once during the week).

#### Theory of planned behavior

All questionnaires assessing the constructs of the TPB were based on guidelines provided by Ajzen and Madden (1986). The questionnaire comprised items on behavioral intention, perceived behavioral control, subjective norm and attitude. Items were rated on a 7-point Likert-type scale with anchors differing for each scale.

***Intention***. Participants responded to three items: “I intend to attend the fitness center on a regular basis for the next 20 weeks” (extremely unlikely—extremely likely), “I will try to…” (definitely true—definitely false), and “I plan to…” (strongly disagree—strongly agree). Cronbach's alpha was 0.85.

***Perceived behavioral control***. In line with Ajzen (2006), perceived behavioral control was measured on the basis of two terms: capability and controllability. Each dimension had two items. The capability items were: “For me to attend regularly…” (impossible—possible) and “If I wanted to I could regularly attend…” (definitely true—definitely false). The controllability items were: “How much control do you believe you have over attending…” (no control—complete control) and “It is mostly up to me whether or not I attend …” (strongly agree—strongly disagree). Cronbach's alpha of the composite scale was 0.74.

***Subjective norm***. Two items measured the injunctive subjective norms: “Most people who are important to me want me to attend …” (definitely true—definitely false) and “Most people who are important to me do not think I should attend…” (strongly agree—strongly disagree). Descriptive subjective norms were assessed using the following two items: “Most people who are important to me will attend …” (definitely true—definitely false) and “The people in my life whose opinions I value attend … (definitely true—definitely false). Cronbach's alpha of the conjoint scale was 0.69.

***Attitude***. In line with Ajzen (2006), the attitude toward regularly attending the fitness center was measured using two items each—two for assessing the instrumental aspects of attitude (worthless—valuable; good—bad) and two for assessing the affective aspects of attitude (pleasant—unpleasant; interesting—boring). The anchors for the 7-point scale were definitively true—definitively false. Cronbach's alpha for the global score was 0.80.

#### Social cognitive theory

***Goals***. Goals were measured using a modified version of a 4-item scale developed by Rise et al. (2003): “I expect to …”; “I intend to …”; “I plan to …”; “How likely is that you ….” 7-point response scales anchored at “very unlikely” (1) and “very likely” (7) were used for all items. We modified it in order to be specific to the context of a fitness center. Cronbach's alpha was 0.87 for this measure.

***Self-efficacy***. Self-efficacy expectations were assessed by the questionnaire developed by Wagner (2000). Subjects were asked to indicate their confidence in their ability to attend the fitness studio regularly in the face of eight potential barriers: physical fatigue, boredom, dejection, visits by friends, family responsibilities, other time-consuming commitments, and work schedule. The scale starts with the statement “I am sure that I will attend the fitness center even if …” (e.g., I am tired). Self-efficacy ratings were obtained along a 5-point scale. Cronbach's alpha was 0.70.

***Outcome expectancies***. Outcome expectancies were assessed using a set of 21 questions developed by Renner et al. (1996) which include the aspects of physical (e.g., decreasing risk of a heart attack, less weight problems), social (e.g., meeting new friends), and self-evaluative (e.g., feeling better) outcome expectancies. The scale starts with the statement “When I regularly attend the fitness center….” Each item represents the second part of this sentence (e.g., “… then I have less weight problems”). Subjects rated their expectations that regular attendance would lead to particular outcomes using a 4-point bipolar scale (definitely true—definitely false). Cronbach's alpha was 0.81.

***Impediments***. The impediments questionnaire assessed environmental barriers (Woll, 2004). Participants were asked to rate the extent to which they agreed or disagreed with six statements asking how much a particular barrier (e.g., distance, sickness, lack of time) interfered with regularly attending the fitness center on a 5-point scale (strongly agree—strongly disagree). The scale started with the statement “When I do not go to the fitness center it is because…” (e.g., “… I am ill”). Cronbach's alpha was 0.68.

***Facilitators***. Social support was assessed using a questionnaire developed by Fuchs (1997). The questionnaire contains six items to social support by family (e.g., a family member encourages me to go to the fitness center) and six items to social support by friends (e.g., friends of mine encourage me to go to the fitness center). It takes instrumental (go with me to the fitness center; take over part of my household chores so that I can go to the fitness center), emotional (encourage me to go to fitness center; invite me to go to fitness center), and organizational (remind me to go to the fitness center; help me to organize my fitness activities) aspects of social support into account. This scale was slightly modified to assess social support for attending the fitness center, at the same time the rating format was changed from a 4-point to a 5-point bipolar scale (completely sure—not sure at all). Cronbach's alpha was 0.80.

#### Physical activity maintenance theory

***Goal-setting***. The questionnaire for personal goals is divided into three sub-dimensions: commitment, attainment, and satisfaction with personal goal attainment. Each sub-dimension consists of ten items. The commitment sub-dimension begins with the sentence “By attending the fitness studio regularly, I am committed to…,” the attainment sub-dimension with “Because I regularly attend a fitness center, I am…,” and the satisfaction sub-dimension with “Because I regularly attend a fitness center, I am satisfied with….” The participants rate the degree of agreement on a 5-point scale (strongly disagree—strongly agree) for each item. According to Nigg et al. (2008), this measure is based on the outcome expectancy measure described by Steinhardt and Dishman (1989) and adapted to the goal-setting for maintenance of PA. Cronbach's alpha was 0.94.

***Self-motivation***. The self-motivation questionnaire is divided into two sections assessing the motivation for PA maintenance with ten items as well as pros and cons of maintaining regular PA with nine items. In the section for motivation for PA maintenance, participants rate statements (e.g., “I'm good at keeping promises, especially the ones I make to myself”) on a 5-point scale (very unlike me—very much like me). Accordingly to Nigg et al. (2008), the sub-scale was developed referring to Dishman et al. (2006) and to Motl et al. (2003). In the section decisional balance, statements related to fitness center attendance (e.g., “I have more energy for my family and friends when I regularly attend a fitness center”) are also rated on a 5-point scale (not at all important—extremely important). Nigg et al. (2008) reported that the scale based on the framework provided by Prochaska et al. (1994). Cronbach's alpha was 0.80.

***Self-efficacy***. According to Nigg et al. (2008), the questionnaire should assess barrier and relapse self-efficacy. The questionnaire comprises 10 items, 6 items for barrier self-efficacy (e.g., “when I have a lot of stress”; “when I am on a journey”) and four items for relapse self-efficacy (e.g., “I will continue to work out even when I previously missed a few sessions”). Participants rated their confidence to regularly attend the fitness center on a 5-point scale (not at all confident—completely confident). Cronbach's alpha was 0.75 for the barrier self-efficacy and 0.71 for the relapse self-efficacy.

***Life stress***. Based on the Recent Life Changes Questionnaire (Miller and Rahe, 1997), a questionnaire with 12 items was developed to indicate the impact of life stress on PA maintenance (Nigg et al., 2008). Participants were asked to rate the impact of different life events (e.g., financial problems, death of a family member) on PA maintenance on a 5-point scale (no impact—high impact). Internal Cronbach's alpha was 0.89.

***Physical activity environment***. According to Nigg et al. (2008), PA environment should comprise physical environment as well as social support. Social support was assessed by the questionnaire developed by Fuchs (1997) and physical environment by the environmental barrier scale developed by Woll (2004). Both scales are described above.

### Statistical analyses

The development of the fitness center attendance over 20 weeks was analyzed using a latent class zero-inflated Poisson growth curve model (Long, 1997) in Mplus (Muthén and Muthén, 2010). The zero-inflated Poisson model is well suited for the analysis of fitness center visits that are time structured count data with an excess of zeros (no shows). In line with prior literature which suggested that exercise participation might well be described by categorical participation patterns (Seelig and Fuchs, 2011), we used latent class analysis to identify these patterns. The number of classes was determined using Akaike Information Criterion (AIC), Bayesian Information Criterion (BIC), and adjusted BIC. In addition, Entropy, the Vuong-Lo-Mendell-Rubin likelihood ratio Test, and the adjusted Vuong-Lo-Mendell-Rubin likelihood ratio Test were computed. TPB, SCT, and PAMT were used to predict class membership. All predictor variables were standardized with a mean of zero and standard deviation of one.

Maximum Likelihood was used for parameter estimation. Because no-shows were analyzed as part of the model, the dependent variable contains no missing values. For the independent variables, the proportion of missing data was 1.08% (range from 0.00 to 3.10% across variables). Parameters and standard errors (SEs) were estimated for initial status (i.e., the latent factor at baseline) and change trajectories (i.e., linear and nonlinear trends).

## Results

### Descriptive results

**Figure 1:**
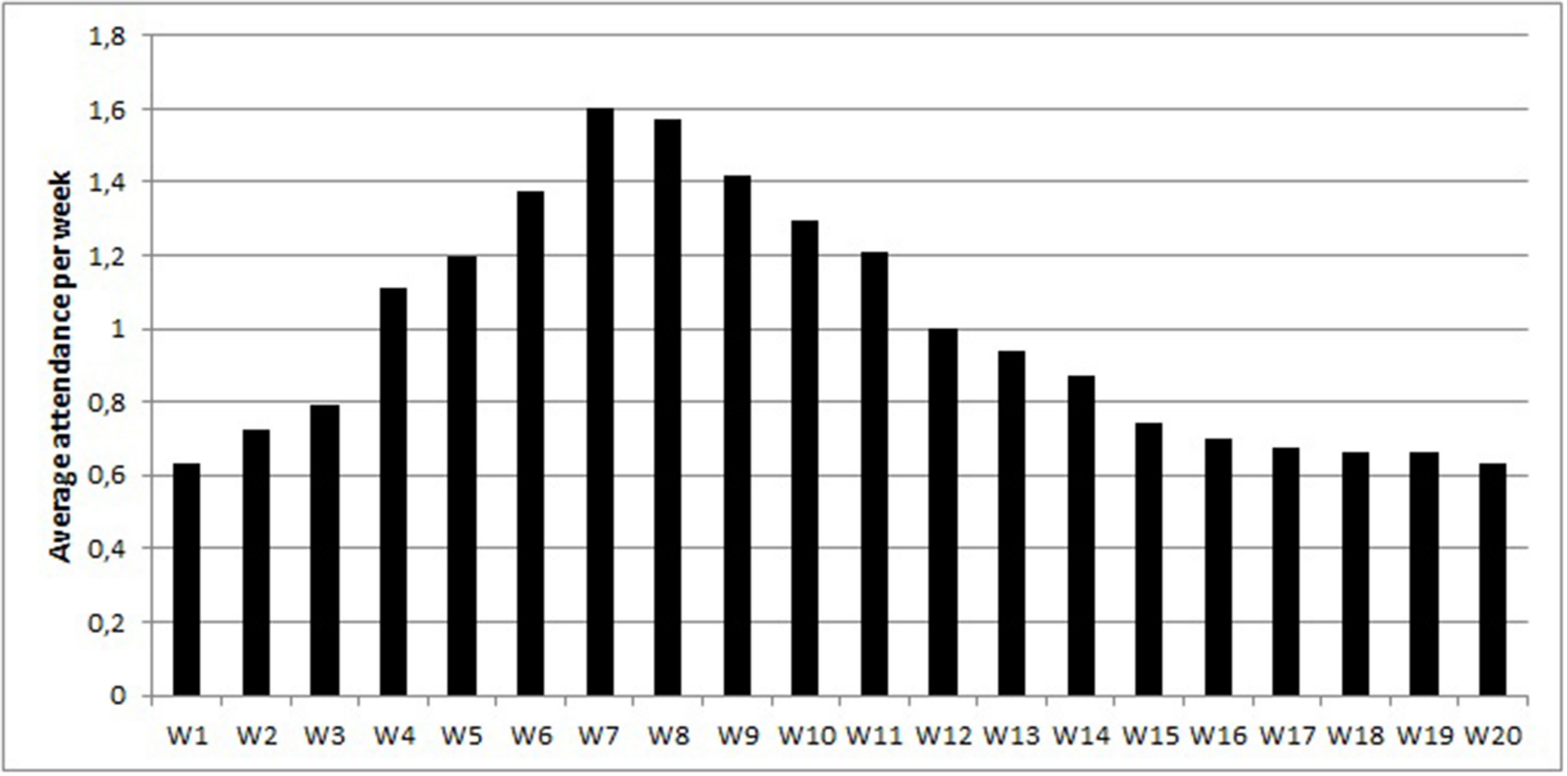
**Average attendance across 20 weeks**.

Figure 1 shows the average exercise participation frequency during the observed 20 weeks, which exhibits a curvilinear trend. Participation frequency was 0.63 during the first week and it reached its maximum in the seventh week with an attendance rate of 1.60. In the last week, the attendance rate returned to 0.63. At all measurement occasions attendance rates significantly differed from zero. Table 1 shows the means, standard deviations, and correlations of individual exercise participation frequency across the 20 weeks and all time-invariant predictors. In the TPB, attitude toward behavior (*r* = 0.21) and perceived behavioral control (*r* = 0.28) were significantly correlated with exercise participation rate. In the SCT, social support by friends (*r* = 0.30) and perceived barriers (*r* = −0.30) were significantly correlated with exercise participation rate. In the PAMT, relapse self-efficacy (*r* = 0.27) and life stress (*r* = −0.21) were significantly correlated with exercise participation rate.

**Table 1:** **Descriptive statistics**.

		**Mean**	***SD***	**2**	**3**	**4**	**5**	**6**	**7**	**8**	**9**
**DEMOGRAPHICS**											
**Regular attenders**											
	Age	24.3	2.7								
	Gender (0 = m; 1 = w)	0.49	0.50								
**Intermittent exercisers**											
	Age	22.9	3.3								
	Gender (0 = m; 1 = w)	0.57	0.49								
**Theory of planned behavior**											
1	Exercise frequency	0.99	0.65	−0.18	0.09	0.21^*^	0.28^**^				
2	Intention	5.35	2.50		−0.09	0.35^**^	0.47^**^				
3	Subjective norm	17.14	3.24			−0.01	−0.13				
4	Attitude	9.20	3.21				0.12				
5	PBC	10.81	3.83								
**Social cognitive theory**											
1	Exercise frequency			0.00	−0.02	−0.11	0.30^**^	−0.30^**^			
2	Self-efficacy	35.92	6.72		0.18	0.12	−0.14	−0.26^**^			
3	Outcome expect.	53.42	7.85			0.11	0.18	0.23^*^			
4	Soc. Support family	10.89	4.45				0.21^*^	0.03			
5	Soc. Support friends	14.17	5.30					0.13			
6	Barriers	15.23	3.48								
**Physical activity maintenance theory**											
1	Exercise frequency			0.08	0.12	0.01	−0.04	0.09	0.16	0.27^**^	−0.21^*^
2	Satisfaction	34.42	5.76		0.88^**^	0.70^**^	0.21^*^	0.57^**^	0.36^**^	0.16	−0.02
3	Attainment	33.93	5.58			0.77^**^	0.25^**^	0.63^**^	0.36^**^	0.13	0.07
4	Commitment	36.30	6.00				0.31^**^	0.58^**^	0.27^**^	0.07	0.08
5	Self-motivation for maintenance	37.31	5.99					0.26^**^	0.40^**^	0.05	−0.08
6	Self-motivation pros/cons	21.19	5.11						0.31^**^	0.20	0.21
7	Self-efficacy barrier	20.01	4.28							0.40	−0.09
8	Self-efficacy relapse	11.44	2.18								−0.14
9	Life stress	5.69	5.97								

SD, standard deviation; PBC, perceived behavioral control; Soc. Support, Social support; Outcome Expect., outcome expectancies;

* p < 0.05;

** *p < 0.01; Gender: men = 0; women = 1*.

### Categorical participation patterns

To identify distinct categorical participation patterns, a latent class zero-inflated Poisson growth curve analysis was carried out. Preliminary analyses showed that a second order polynomial growth curve model provided a good description of the participation rate over the 20 weeks. Based on this preliminary analysis, three different models were estimated in which the variance of the intercept, linear slope, and, quadratic slope in the zero-part, as well as in the count-part, were constrained to zero: a one-class model (1), a two-class model (2), and a three-class model (3). Model comparisons are presented in Table 2. Fit indices indicated an improvement in model fit from the one-class model (AIC = 3509.4; BIC = 3525.1; sample adjusted BIC = 3506.1) to the two-class model (AIC = 3212.7; BIC = 3246.7; sample adjusted BIC = 3205.6). In line with the information criteria, the Vuong-Lo-Mendell-Rubin Test (value = 310.7; *p* < 0.01), as well as the adjusted Lo-Mendell-Rubin Test (value = 301.4; *p* < 0.01), indicated a significant improvement in model fit. For the three-class model, there was a further slight improvement in AIC (AIC = 3150.0) and BIC (BIC = 3202.2; sample adjusted BIC = 3139.2). However, neither the Vuong-Lo-Mendell-Rubin Test (value = 76.6; *p* = 0.52) nor the adjusted Lo-Mendell-Rubin Test (value = 74.3; *p* = 0.52) suggested a significant improvement. Furthermore, entropy of the two-class model (0.923) was higher than that of the three-class model (0.904) indicating a better model fit for the two-class model. As a result, the two-class model was used for all subsequent analyses.

**Table 2:** **Comparison of the one-class, two-class, and three-class models**.

	**1 Class (1)**	**2 Classes (2)**	**3 Classes (3)**
Free parameters	6	13	20
AIC	3509.40	3212.68	3150.05
BIC	3525.10	3246.67	3202.25
Sample adjusted BIC	3506.14	3205.61	3139.18
Entropy		0.92	0.90
Vuong, Lo, Mendell, Rubin	n.a.		
Model test		2 v 1	3 v 2
−2LL difference		310.73	76.63
*p*		0.00	0.52
Lo, Mendel, Rubin adjusted	n.a.		
Model test		2 v 1	3 v 2
−2LL difference	n.a.	301.40	74.33
*p*	n.a.	0.00	0.52
*N* for each class	c1 = 101	c1 = 46;	c1 = 37;
		c2 = 55	c2 = 20;
			c3 = 44

*AIC, Akaike Information Criterion; BIC, Bayesian Information Criterion; -2LL, -2 Log Likelihood; p, Probability Value; N, number of participants*.

The estimates for the latent class zero-inflated Poisson growth curve model for the two-class solution is presented in Table 3. For Class 1, the attendance rate for the first week is 1.55 time per week, which differs significantly from zero. Linear and quadratic trajectories of the participation rate do not significantly deviate from zero indicating that the participants, in this class, attended the fitness center at about the same level over 20 weeks. The probability of not attending the fitness center in the first week is 33%. With a value of −0.57, the quadratic trajectory of the likelihood for not attending the fitness center is significantly lower than zero, suggesting an inverted-u function for the likelihood of non-attendance. Participants in this class can be interpreted as regular attenders.

**Table 3:** **Model estimates of the latent class zero-inflated poisson growth curve model for the two-class solution**.

	**Estimate**	***SE***	**Estimate/*SE***	***p***	**Odd**	**Prob. of no attendance**
**CLASS 1**						
**Participation**						
Intercept	1.55	0.11	5.19	0.00		
Lin traj	0.02	0.02	0.67	0.51		
Quadr. traj.	0.00	0.00	−1.32	0.19		
**Probability of not attending**						
Intercept	−0.70	0.43	−1.63	0.10	0.50	0.33
Lin. traj.	0.85	0.48	1.76	0.08		
Quadr. traj.	−0.57	0.18	−3.10	0.00		
**CLASS 2**						
**Participation**						
Intercept	−0.24	0.22	−1.09	0.28		
Lin. traj.	0.21	0.07	2.97	0.00		
Quadr. traj.	−0.02	0.01	−4.14	0.00		
**Probability of not attending**						
Intercept	1.01	0.49	2.07	0.04	2.73	0.73
Lin. traj.	−0.01	0.28	−0.04	0.97		
Quadr. traj.	−0.12	0.05	−2.51	0.01		

*SE, standard error; Lin. traj., linear trajectory; Quadr. traj., quadratic trajectory*.

For Class 2, the attendance rate for the first week is not significantly different from zero. However, both the linear and quadratic trajectories are significant, indicating that the participants' attendance rate changes over the consecutive 20 weeks. In the first week, the attendance rate is very low but increases continuously in the consecutive weeks. The maximum is reached in the seventh week, after that the attendance rate only decreases continuously. The probability of not attending the fitness center in the first week is 73%. The quadratic trajectory of the likelihood for not attending the fitness center is with a value of 0.12, significantly different from zero, suggesting a u-shaped function for the likelihood of non-attendance. The participants in this class can be seen as intermittent exercisers.

### Predicting the class membership

Class membership was predicted using logistic regression analysis. The same two-class-model as described above was applied separately for all three prediction theories. For each theory, a similar developmental pattern of participation was found in both classes. In the class of regular attenders, the intercept differed significantly from zero and non-significant linear and quadratic trajectories of participation rate were observed. In the class of intermittent exercisers, significant linear and quadratic trajectories for participation rate were found.

#### Theory of planned behavior

In the logistic regression presented in Table 4, two variables of the TPB significantly predicted class membership: attitude toward behavior and perceived behavioral control. An increase of one standard deviation in the variable attitude toward behavior leads to an increasing likelihood to belong to the class of regular attenders by 1.52. Equivalently, an increase of one standard deviation in the variable perceived behavioral control increased the odds to be in the class of regular attenders by 1.74 (Nagelkerke's pseudo *R*^2^ = 0.17).

**Table 4:** **Model estimates of the latent class zero-inflated poisson growth curve model for the two-class solution**.

	**Estimate**	***SE***	**Estimate/*SE***	***p***	**Odds ratio**
**THEORY OF PLANNED BEHAVIOR**					
Intercept	0.25	0.24	1.06	0.15	
Intention	−0.26	0.70	−0.94	0.83	0.77
Subj. norm	−0.27	0.23	−1.2	0.88	0.76
Attitude	0.42	0.24	1.76	0.04	1.52
PBC	0.55	0.26	2.14	0.02	1.74
Nagelkerke's pseudo *R*^2^ = 0.17					
**SOCIAL COGNITIVE THEORY**					
Intercept	0.25	0.24	1.02	0.16	
Goals	0.32	0.24	1.31	0.10	1.38
Self-efficacy	0.47	0.28	1.66	0.04	1.61
Outcome expectancies	−0.11	0.24	−0.29	0.67	0.90
Barriers	−0.85	0.28	−2.96	0.00	0.43
Social support family	−0.07	0.24	−0.29	0.62	0.93
Social support friends	0.47	0.25	1.87	0.03	1.60
Nagelkerke's pseudo *R*^2^ = 0.28					
**PHYSICAL ACTIVITY MAINTENANCE THEORY**					
Intercept	−0.43	0.29	−1.51	0.07	
Commitment	−0.25	0.36	−0.70	0.75	0.78
Attainment	0.92	0.59	1.56	0.06	2.50
Satisfaction	−0.67	0.54	−1.25	0.89	0.51
Self-motivation PA maint.	−0.16	0.27	−0.58	0.72	0.86
Self-motivation pros/cons	0.14	0.32	0.44	0.33	1.15
Self-efficacy barrier	0.27	0.30	0.89	0.19	1.31
Self-efficacy relapse	0.24	0.26	0.90	0.38	1.27
Life stress	−0.92	0.36	−2.54	0.01	0.40
Nagelkerke's pseudo *R*^2^ = 0.25					

*SE, standard error; p, probability value; Subj. norm, subjective norm; PBC, perceived behavioral control; Self-motivation PA maint., motivation for physical activity maintenance*.

#### Social cognitive theory

The results of the logistic regression (see Table 4) indicated that three variables of the SCT significantly predicted class membership: self-efficacy, perceived barriers, and social support by friends. Increasing self-efficacy expectations by one standard deviation increased the odds of belonging to the class of regular attenders by 1.61. An increase of one standard deviation in the social support by friends increased the odds of belonging to the class of regular attenders by 1.60. Finally, increasing the rating in perceived barriers by one standard deviation decreased the odds of belonging to the class of regular attenders by 2.3 (=1/0.43) (Nagelkerke's pseudo *R*^2^ = 0.28).

#### Physical activity maintenance theory

In the PAMT, only one variable significantly predicted membership in both classes: life stress. Increasing perceived life stress by one standard deviation led to a decrease in the odds of being a member of the class of regular attenders by 2.5 (=1/0.40) (Nagelkerke's pseudo *R*^2^ = 0.25).

## Discussion

TPB as well as SCT are among the most widely used theoretical frameworks to explain and predict exercise behavior. Considerably less attention was devoted to the PAMT, which was explicitly developed to explain PA maintenance. The purposes of the present study were (i) to describe the development of PA participation over time, (ii) to predict development of PA participation by the TPB, SCT, and PAMT, and (iii) to compare the predictive power of the three theories.

In order to describe the development of exercise behavior over 20 weeks a latent class zero-inflated Poisson growth curve analysis was carried out. The procedure identified two different participation patterns. In the class of regular attenders, participants exercised on average 1.55 times in the first week with a probability of not attending the fitness center of 33%. In the 19 following weeks, their participation rate did not significantly change indicating that they maintained their physical activity at a comparable level. On the contrary, strong fluctuations in exercise behavior were observed in the class of intermittent exercisers. At the beginning of the semester, their participation rate was not different from zero with a probability of not attending the fitness center of 73%. However, they increased their attendance rate during the semester reaching a maximum in the middle of the semester (i.e., the seventh week). Thereafter, their participation rate decreased continuously and the probability of no attendance increased at the same time. At the beginning of the semester, the students probably dispose of more time to devote to exercise and can therefore increase their attendance rate until the middle of semester. However, as the end of the semester approaches and the exam stress increases, the intermittent exercisers fail to exercise continuously.

The difference between these two behavior patterns were explained in terms of three theories: for the TPB, it could be shown that the regular participation pattern was associated with a positive attitude toward attending the fitness center and higher perceived behavioral control. However, these results are not fully in line with the assumptions of the TPB because behavioral intention—as an immediate and most important predictor—could not differentiate between regular attenders and intermittent exercisers. These results are consistent with findings of the study of Armitage (2005) in which perceived behavioral control, but not behavioral intention, was predictive of PA maintenance. The predictive power of the TPB was quite small with a Nagelkerke's *R*^2^ of 0.17 and can be compared to findings of other studies predicting PA maintenance by the TPB (e.g., Bryan and Rocheleau, 2002; Armitage, 2005).

In the SCT, three predictors were identified to significantly predict exercise behavior. In accordance with several studies on maintenance (Plotnikoff et al., 2008; Anderson et al., 2010), higher perceived self-efficacy was associated with a regular exercise behavior over 20 weeks. Intermittent exercisers perceived more barriers, which might explain their intermittent exercise pattern. Furthermore, perceived social support by friends was higher in regular attenders. These results indicate that higher social support and self-efficacy might be important aspects of regular exercise behavior whereas perceived barriers might result in fluctuations in exercise. Interestingly, outcome expectancies as well as goals were not significant predictors. With a Nagelkerke's *R*^2^ of 0.28, the predictive power is consistent with findings of other studies.

PAMT is a rather new theory and was specifically developed to explain maintenance in PA behavior. The only significant predictor in the PAMT is life stress indicating that participants with high levels of life stress have higher probability to belong to the group of intermittent exercisers. Life stress might lead to distractions from regular PA behavior and impede the habituation process. However, all other predictors such as goal setting, self-motivation, and self-efficacy were not significant predictors of the exercise attendance. The variables of this theory are considerably correlated with each other showing a Nagelkerke's *R*^2^ of 0.25.

When comparing the three theories, SCT had the greatest predictive power with Nagelkerke's *R*^2^ of 0.28 followed by PAMT (Nagelkerke's *R*^2^ = 0.25), whereas TPB showed the lowest predictive power (Nagelkerke's *R*^2^ = 0.17). These results are in line with findings of other studies predicting PA by SCT and TPB where the amount of explained variance was about 20% (Amireault et al., 2013). SCT seems to include predictors with the highest predictive power. However, to motivate large parts of the population to exercise more regularly, stronger prediction models would be needed. An extension of the existing models seems to be a consequence of increasing the predictability of PA maintenance (Rothman, 2000). For example, for the TPB, past behavior, salience beliefs, moral norms, self-identity, and affective variables have been proposed (Conner and Armitage, 1998). This list could be enlarged for barriers and other external impeding variable.

The results of this study suggest that impeding aspects such as life stress and barriers might have the greatest predictive power in the process of exercise maintenance. In the SCT as well as in PAMT, impeding aspects were the strongest predictors of exercise patterns. Presumably, such external factors play a more important role in the process of PA maintenance than predicted by the presented models. Several studies have shown that barriers might be associated with sedentary lifestyle and prevent exercise maintenance (e.g., Salmon et al., 2003; Rimmer et al., 2004). Efficacy expectancies as perceived behavioral control in the TPB and self-efficacy in the SCT were significant predictors in the behavioral maintenance process. In contrast to these findings, relapse and barrier self-efficacies were not predictive of the exercise pattern. Obviously, the differentiation between barrier and relapse self-efficacy did provide additional predictive power with regards to explaining the differences between regular attenders and intermittent exercisers. However, further studies will be needed to explicitly test this hypothesis.

Additionally, when compared to social support from family members, social support from friends might be more important for college students as they normally leave their parental home for studying and have not yet started their own families. Interestingly, subjective norms in the TPB did not contribute to the prediction of exercise attendance. It could be shown consistently for all three theories that goal setting and intentions were not predictive of exercise pattern. It might be that all participants who start to exercise have intentions to exercise regularly but the intention changes over a short period of time as shown by Conroy et al. (2013). However, other motivational or even emotional aspects might be more important in the process of exercise maintenance. The same constellation of predictors was identified in the study by Wen et al. (2003) where self-efficacy, social support, und barriers significantly predicted exercise behavior.

This study has a number of limitations. First, the sample size (101 participants) is relatively small to detect more complex participation patterns. Possibly, a greater sample size would facilitate identification of more detailed participation patterns. Second, predictor variables were measured only at baseline, meaning that week-by-week changes of predictors were not assessed. Third, the sample of the study is highly selective as it contains only college students. This aspect might impair the generalizability of this study. Forth, some scales showed only sufficient internal consistency. This aspect could negatively affect the analyzed effects.

Nonetheless, this study has several merits. First, to our knowledge, the present study is the first study to empirically evaluate PAMT and to compare it to SCT, and TPB in the context of exercise attendance. The dependent variable attendance (rate) was objectively measured by a magnetic card system, which is a reliable and valid assessment of exercise attendance. Finally, sound statistical methods as zero-inflated Poisson growth curve analyses were used to describe participation patterns, which are more informative than the simple prediction of the total number of fitness center attendances.

## Conclusion

This study identified two patterns of exercise attendance: regular attenders and intermittent exercisers. SCT showed greatest predictive power compared to PAMT and TPB. Our hypothesis that the more specified PAMT would predict the exercise patterns more precisely than the rather global theories could not be confirmed. Impeding aspects such as life stress and barriers were the strongest predictors of the participation patterns suggesting that overcoming barriers might be an important aspect in the process of exercise maintenance. Future studies should examine PA maintenance and put stronger focus on these impeding external variables. Self-efficacy, perceived behavioral control and social support were shown to differentiate between regular attenders and intermittent exercisers whereas intentions and goals were not significant predictors. Due to the modest predictive power of all three theories an extension of the theories seems inevitable. Accordingly, the past behavior or affective determinants of behavior, among others, should be taken into account.

### Conflict of interest statement

The authors declare that the research was conducted in the absence of any commercial or financial relationships that could be construed as a potential conflict of interest.
